# Supplementary material for: Revealing single-neuron and network-activity interaction by combining high-density microelectrode array and optogenetics
Source: Nat Commun. 2024 Nov 11;15:9547. doi: 10.1038/s41467-024-53505-w (PMC11555060; doi:10.1038/s41467-024-53505-w)
Supplement: Supplementary file 1 — Supplementary Information [file 41467_2024_53505_MOESM1_ESM.pdf]

Electronic supplementary information

Revealing single neuron and network-activity interaction by combining high-density microelectrode array and optogenetics

Authors: Toki Kobayashi[1*], Kenta Shimba[2*], Taiyo Narumi[2], Takahiro Asahina[3], Kiyoshi Kotani[2],

Yasuhiko Jimbo[1]

[1] Department of Precision Engineering, School of Engineering, The University of Tokyo, Tokyo, Japan
[2] Department of Human and Engineered Environmental Studies, Graduate School of Frontier Sciences, The University of Tokyo, Chiba, Japan
[3] Center for Information and Neural Networks, National Institute of Information and Communications Technology, Osaka, Japan

* Corresponding authors:
Toki Kobayashi, kobayashi.toki.jb@gmail.com
Kenta Shimba, (ORCID: 0000-0003-1156-260X) shimba@neuron.t.u-tokyo.ac.jp

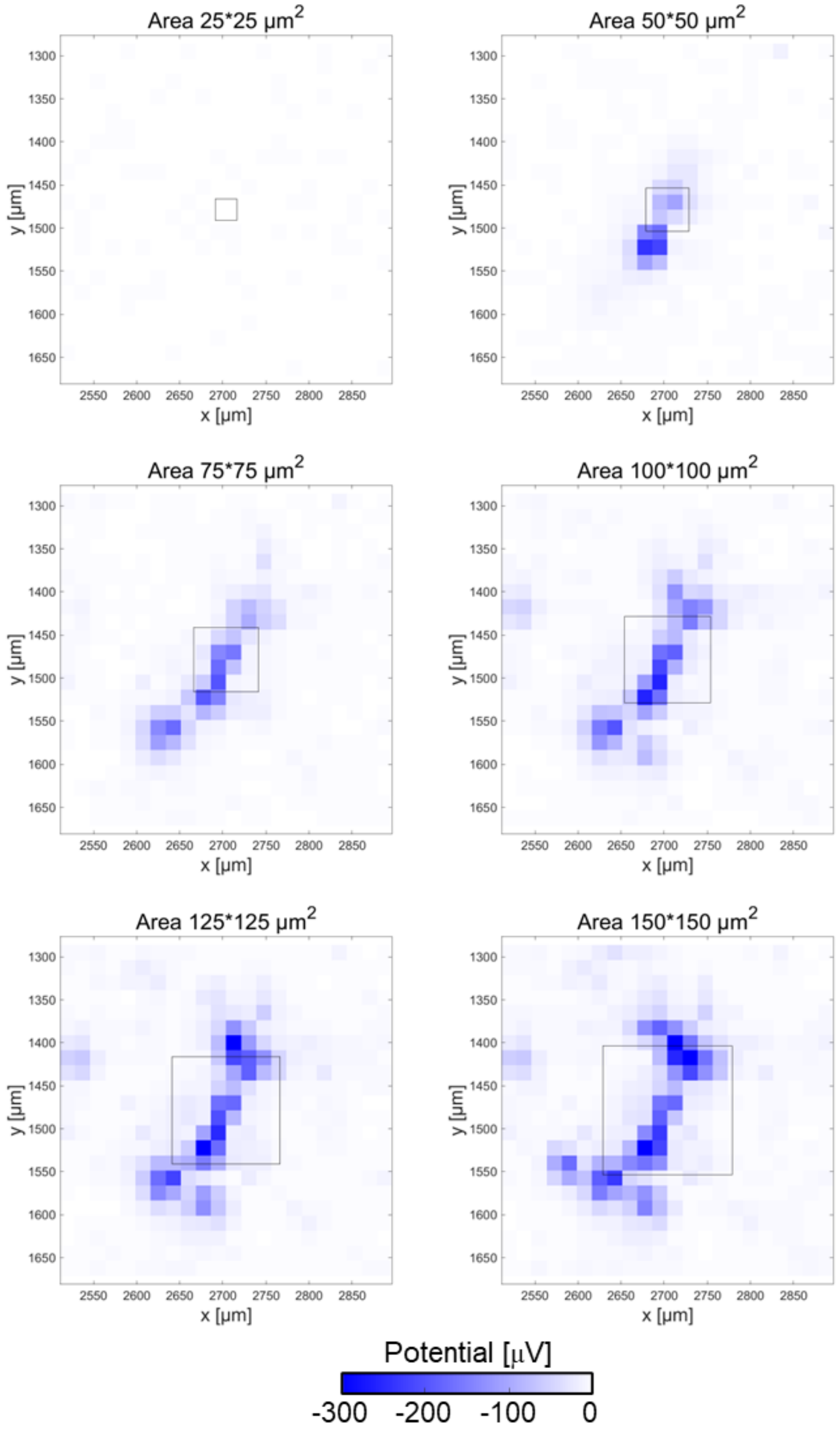

Supplementary Fig. 1. Minimum potential heatmap of stimulus-time-triggered potential signals during stimulation duration (5 ms). The stimulation area was changed and the number of responding cells was evaluated. The stimulation area is bounded by the black-outlined square. The number of peaks corresponds to number the of cells that responded during the stimulation time.

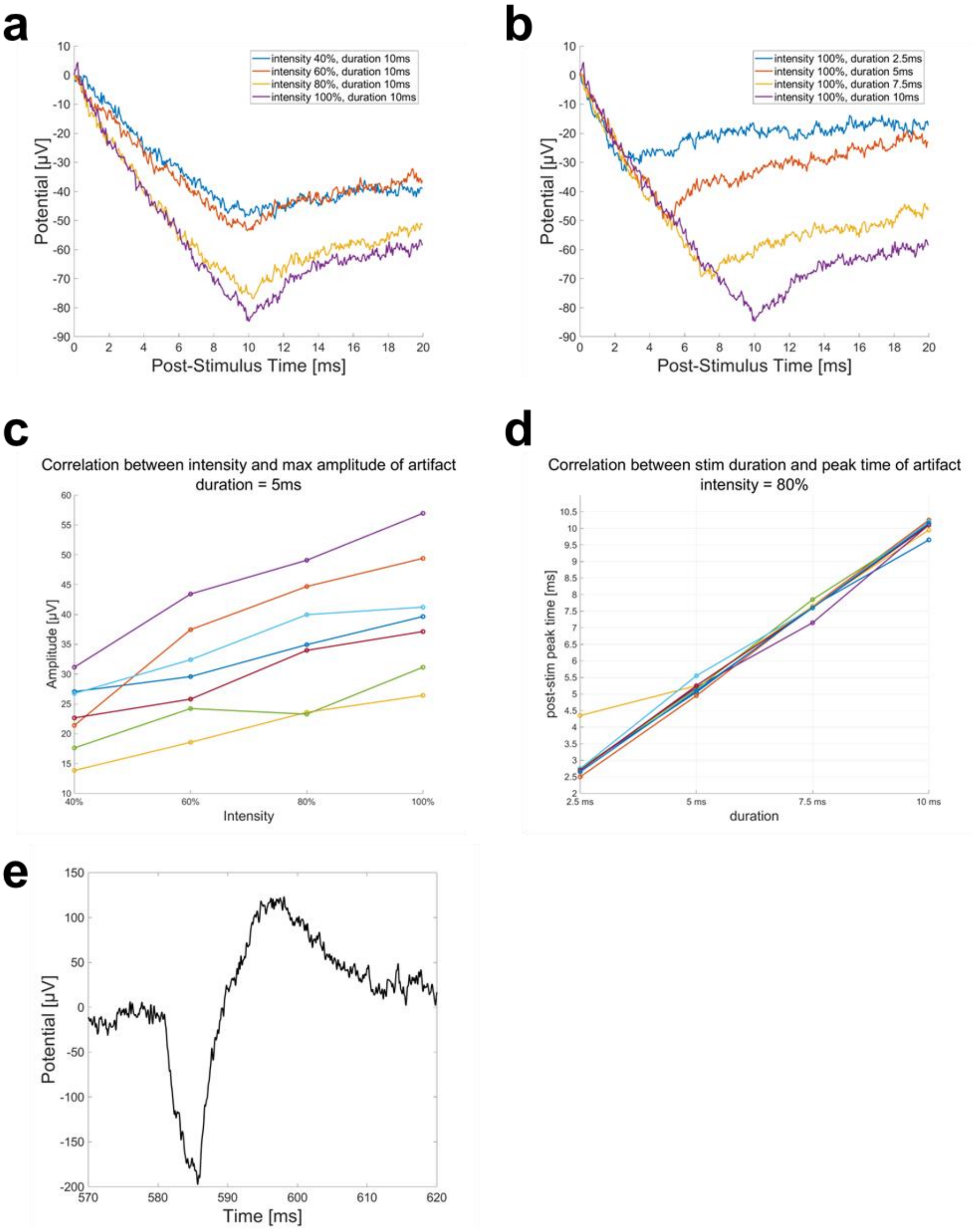

Supplementary Fig. 2. Evaluation of optical-stimulation artifacts. Artifacts were recorded when the intensity, duration, and area of stimulation were changed. (a) The variation in stimulus-time-triggered averaging signals at one electrode when stimulation intensity was changed. (b) The variation in stimulus-time-triggered averaging signals at one electrode when stimulation duration was changed. (c) Relationship between light intensity and artifact size recorded at nine electrodes. (d) Relationship between optical stimulation time and artifact size recorded at nine electrodes. (e) An artifact recorded at one electrode when the entire electrode was stimulated.

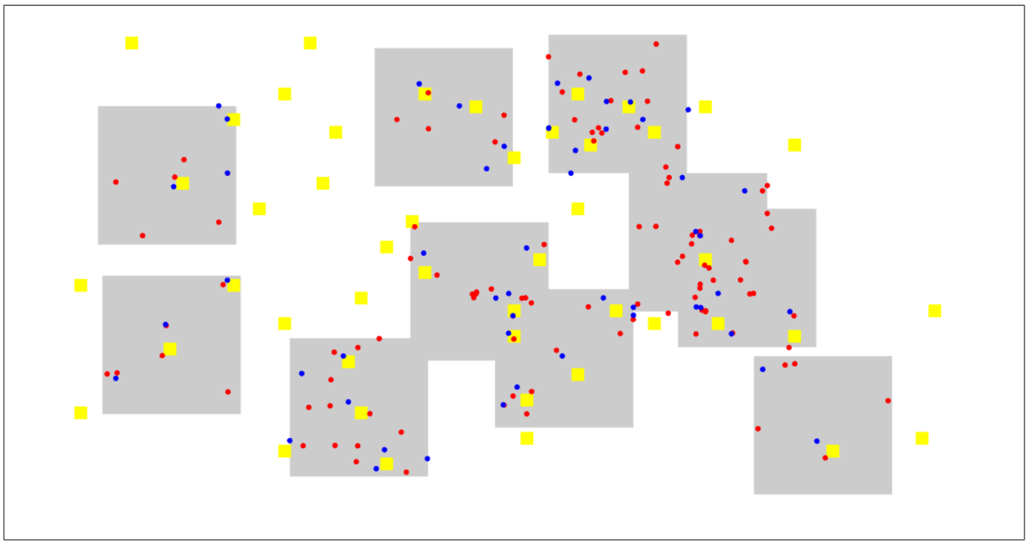

Supplementary Fig. 3. Locations of recording area, optical stimulations, and directly/indirectly responding neurons. Gray shades show the recording area. Yellow squares show the optical stimulation site. Red and blue dots indicate locations of indirectly and directly responding neurons.

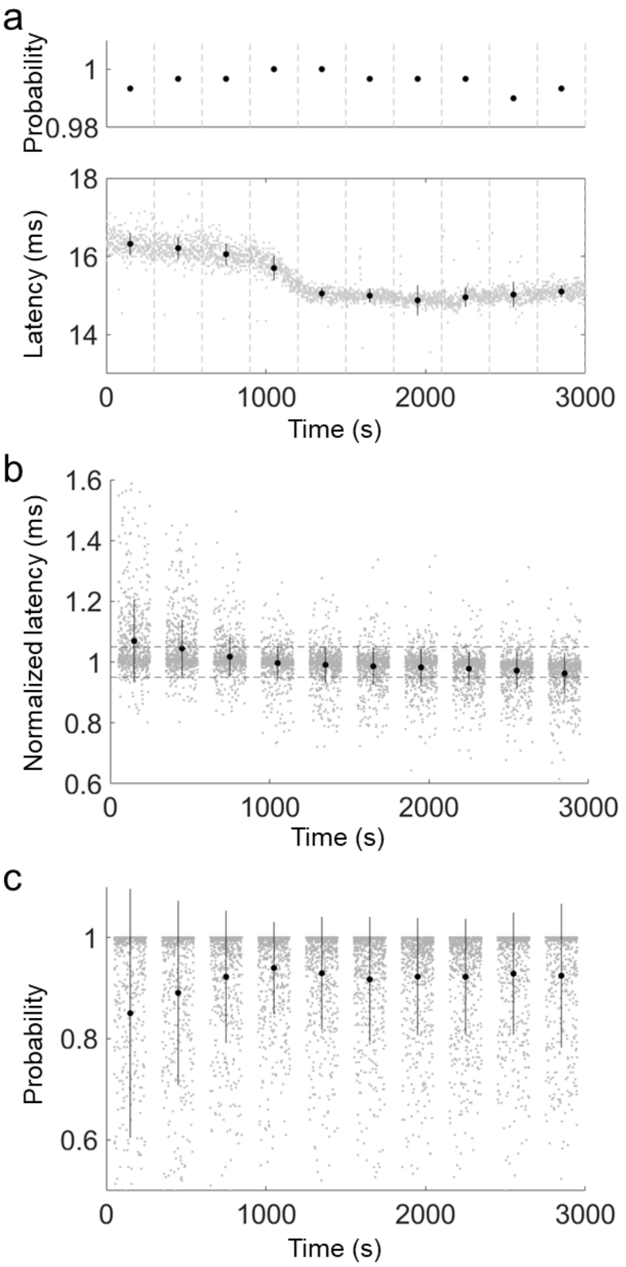

Supplementary Fig. 4. Change in response latency and probability.

(a) Time dependent change in response latency and probability for a representative sample. The latency decreased during the recording period. (b) Change in normalized response latency of all neurons. Latency was averaged every 300 s and normalized the overall average value at each neuron. Horizontal dashed lines show the average ± 5%. Mean ± standard deviation. (c) Change in response probability. Response latency decreased and response probability increased for 5-10 min after the stimulus was initiated.

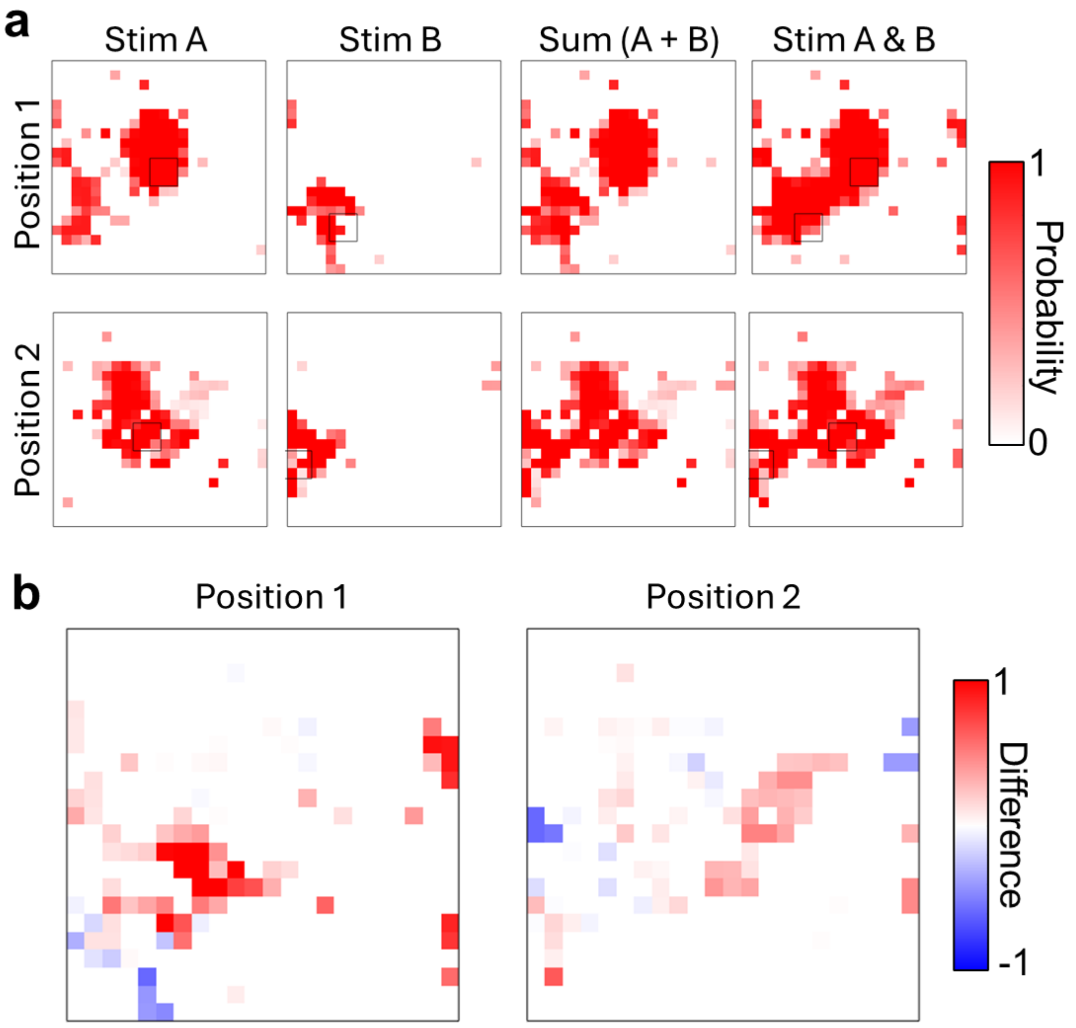

Supplementary Fig. 5. Response probability of single-site and combined stimulation. The single-site stimulations at sites A and B and combined stimulation at both sites A and B were delivered sequentially. (a) Firing probability to stimulus with four different conditions. The figures show the results for, from left to right, response to stimulus A, stimulus B, aggregate response obtained by summing the responses of stimulus A and B, and combined stimulus A+B. (b) Difference between aggregate response and combined stimulation. Red color in (b) shows the response probability was higher with combined stimulation than aggregate response of stimulus A and B. Note that a few electrodes show a decrease in firing probability with the combined stimulus.

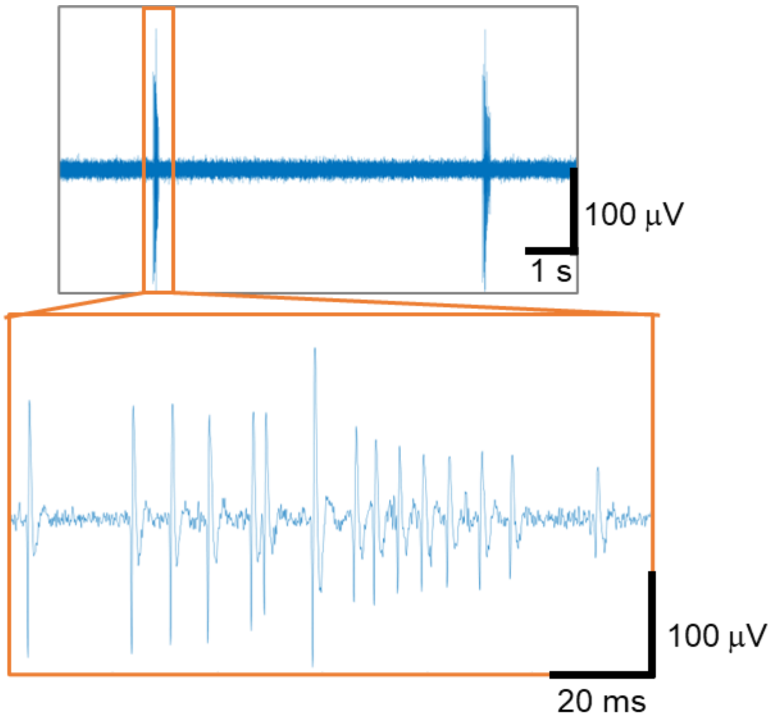

Supplementary Fig. 6. Spontaneous extracellular signal of the leader neuron.
Top: extracellular signal of spontaneous activity in a leader neuron. Bottom: enlarged figure of top.

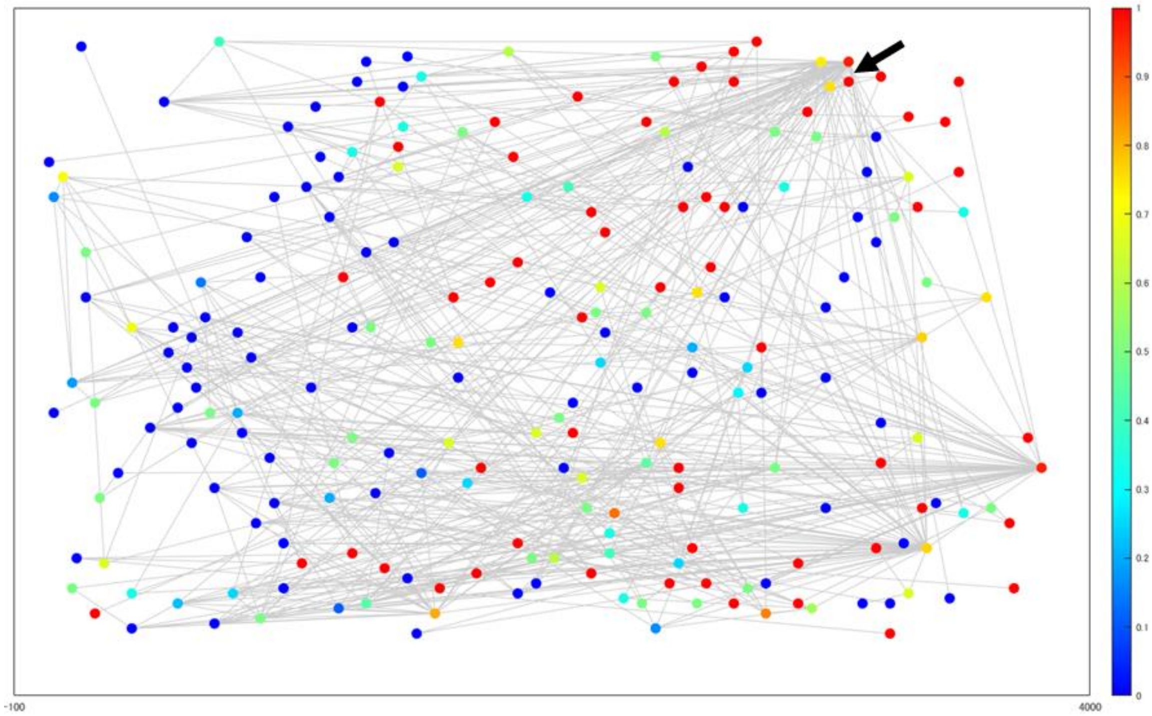

Supplementary Fig. 7. Map of Functional connectivity. Pair-wise functional connectivity was calculated for all pairs of electrodes. Information sender and receiver was defined with the direction of functional connection. Mean ratio of sender and receiver roles was indicated with color; warm color means sender, cold color means receiver. The black arrow indicates the position of the leader neuron, which works as an information sender in the network.

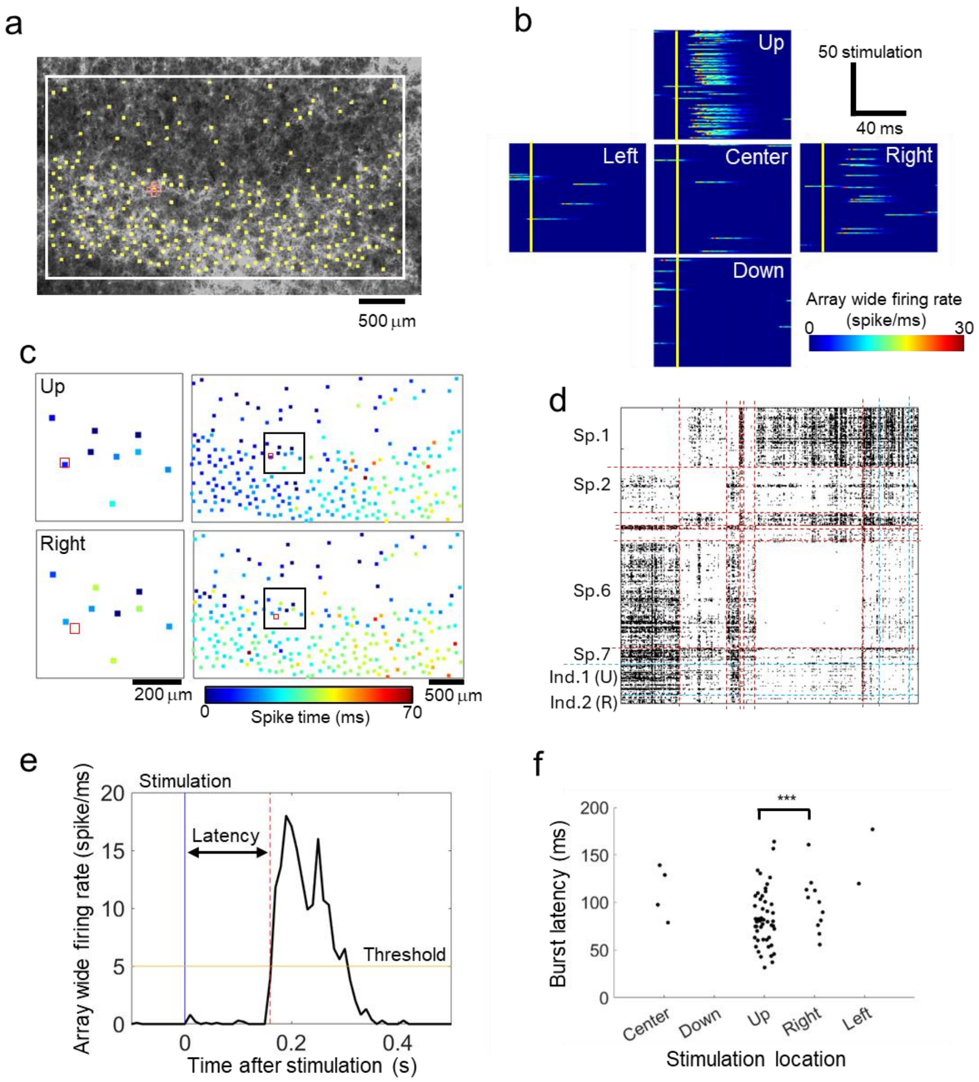

Supplementary Fig. 8. Relationship between stimulation location and leader neurons. (a) Locations of recording electrodes and stimulation. Optical stimulation was applied to five adjacent areas shown by the red square (target plus one square shifted up, down, left, and right). Electrodes are shown with yellow squares. (b) Firing rate after optical stimulation. Network bursts were induced by optical stimulation to Up location. Yellow vertical lines show the stimulation time. (c) Burst propagation patterns induced by optical stimulation to Up and Right locations. The overall pattern of propagation was similar (right panels), but the response time was different for the electrodes closer to the stimulus locations (left panels). Red squares indicate stimulation locations. (d) Similarity matrix of burst propagation pattern. Induced bursts from Up location (Ind. 1) and Right location (Ind.2) are significantly similar to Spontaneous burst 6 (Sp.6). (e) Calculation method for latency of network burst. (f) Comparison among burst latency. Latency of network bursts induced by optical stimulation to Up location was significantly shorter than that to Right

location. ***, $p < 0.001$; Mann–Whitney U test; n = 49 bursts from Up location, n = 11 bursts from Right location.

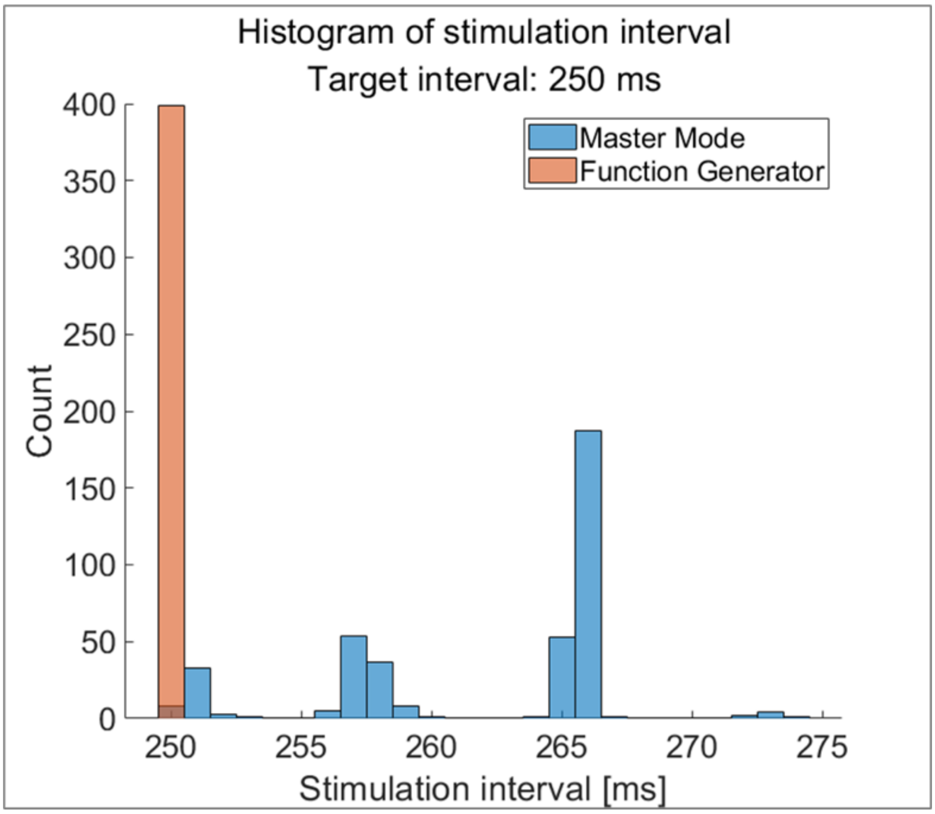

Supplementary Fig. 9. Histogram of stimulation interval under two control methods. Blue: Stimulus interval of Polygon1000-G controlled by Polyscan2 (Master Mode). Orange: Stimulus interval of Polygon1000-G controlled by an external signal from a function generator. The stimulation interval was set to 250 ms. Stimulation was applied 400 times.

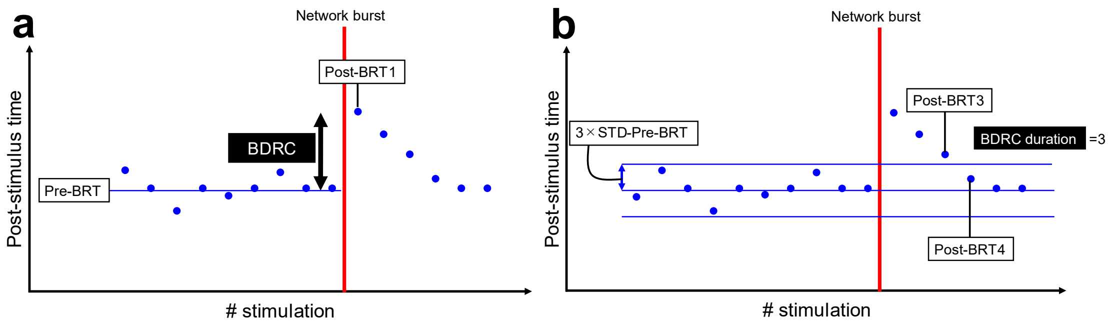

Supplementary Fig. 10. Analysis method for quantification of the BDRC size and duration. (a) Quantification of BDRC. (b) Quantification of BDRC duration.

Supplementary Table 1 Equipment list: Experimental system for optical stimulation and electrical recording.

Name	Vender	Catalog number	Other
Polygon 1000-G	Mightex Systems, Ontario, Canada	DSI-K2-000	
3-Position adaptor	Mightex Systems, Ontario, Canada	DSI-3PS-OL-UA-PY	For Olympus BX series
Beam splitter	Mightex Systems, Ontario, Canada	DSI-BS-90R-10T-UF1	90% reflection, 10% through
LED light source 470 nm	Mightex Systems, Ontario, Canada	LCS-0470-50-22	
LED light source 560 nm	Mightex Systems, Ontario, Canada	LCS-0560-68-22	
LED controller	Mightex Systems, Ontario, Canada	BLS-13000-1E	For 470 nm LED
LED controller	Mightex Systems, Ontario, Canada	BLS-18000-1	For 560 nm LED
A/D input-output control module	Mightex Systems, Ontario, Canada	BLS-IO04-US	
Beam combiner	Mightex Systems, Ontario, Canada	LCS-BC25-0505	
Light guide adaptor	Mightex Systems, Ontario, Canada	LCS-LGA-0515	
Liquid light guide	Mightex Systems, Ontario, Canada	LLG-03-59-340-0800-1	Ferrule diameter 5 mm
Upright microscope	Olympus, Tokyo, Japan	BX51WI	
Mirror units	Olympus, Tokyo, Japan	U-MNIBA3	
Mercury lamp	Olympus, Tokyo, Japan	USH-103OL	
Microscope camera	Wraymer, Osaka, Japan	WRAYCAM-VEX230M	
MaxOne High-Density Microelectrode Array System	MaxWell Biosystems AG, Zurich, Switzerland	-	
Mini Air Pump EAP-01	As One Corp., Osaka, Japan	1-7578-01	Suction-Discharge Convertible Type
Function Generator AWG1005	As One Corp., Osaka, Japan	3-6696-01	
Objective lens	Evident Corp., Tokyo, Japan	MPLFLN2.5x	
3D printer Form3	Formlabs Japan, Tokyo, Japan	Form3	For recording unit holder and chip lids
Thermostat	Omron Corp., Kyoto, Japan	E5CN	
Vibration isolator	Meiritz Seiki Co., Ltd., Kanagawa, Japan	AVT-0405N	
Acrylic box	-	-	Custom-made<br>CAD data will be provided upon request.
