# Supplementary material for: Revealing single-neuron and network-activity interaction by combining high-density microelectrode array and optogenetics
Source: Nat Commun. 2024 Nov 11;15:9547. doi: 10.1038/s41467-024-53505-w (PMC11555060; doi:10.1038/s41467-024-53505-w)
Supplement: Supplementary file 3 — Description of Additional Supplementary Files [file 41467_2024_53505_MOESM3_ESM.pdf]

Description of additional Supplementary Files

Supplementary Movies 1.

An example of signal propagation from a directly responding neuron to an indirectly responding neuron. Extracellular potentials were overlapped on an immunofluorescence image.

Supplementary Movies 2.

An example of indirect responses of an integrator neuron. three different neurons directly responded to a specific stimulus, while one neuron consistently showed indirect responses to three different stimuli.

Supplementary Movies 3. An example of network bursts initiated by an leader neuron. The leader neurons was stimulated with 5-s interval.

Supplementary Code 1 .
