# Supplementary material for: Revealing single-neuron and network-activity interaction by combining high-density microelectrode array and optogenetics
Source: Nat Commun. 2024 Nov 11;15:9547. doi: 10.1038/s41467-024-53505-w (PMC11555060; doi:10.1038/s41467-024-53505-w)
Supplement: Supplementary file 8 — Reporting Summary [file 41467_2024_53505_MOESM8_ESM.pdf]

nature portfolio

Corresponding author(s): Kenta Shimba

Last updated by author(s): Oct 6, 2024

Reporting Summary

Nature Portfolio wishes to improve the reproducibility of the work that we publish. This form provides structure for consistency and transparency in reporting. For further information on Nature Portfolio policies, see our Editorial Policies and the Editorial Policy Checklist.

Statistics

For all statistical analyses, confirm that the following items are present in the figure legend, table legend, main text, or Methods section.

n/a	Confirmed	
☐	☒	The exact sample size (n) for each experimental group/condition, given as a discrete number and unit of measurement
☐	☒	A statement on whether measurements were taken from distinct samples or whether the same sample was measured repeatedly
☐	☒	The statistical test(s) used AND whether they are one- or two-sided<br>*Only common tests should be described solely by name; describe more complex techniques in the Methods section.*
☒	☐	A description of all covariates tested
☒	☐	A description of any assumptions or corrections, such as tests of normality and adjustment for multiple comparisons
☐	☒	A full description of the statistical parameters including central tendency (e.g. means) or other basic estimates (e.g. regression coefficient) AND variation (e.g. standard deviation) or associated estimates of uncertainty (e.g. confidence intervals)
☐	☒	For null hypothesis testing, the test statistic (e.g. F, t, r) with confidence intervals, effect sizes, degrees of freedom and P value noted<br>*Give P values as exact values whenever suitable.*
☒	☐	For Bayesian analysis, information on the choice of priors and Markov chain Monte Carlo settings
☒	☐	For hierarchical and complex designs, identification of the appropriate level for tests and full reporting of outcomes
☒	☐	Estimates of effect sizes (e.g. Cohen's d, Pearson's r), indicating how they were calculated

Our web collection on statistics for biologists contains articles on many of the points above.

Software and code

Policy information about availability of computer code

Data collection	MaxLab Live (Maxwell Biosystems, Zurich, Switzerland) for recording extracellular signals<br>PolyScan2 (Mightex, CA, USA) for controlling DMD device<br>MicroStudio, x64, 1.4.17015.20200426 (Wraymer, Osaka, Japan) for taking GFP fluorescence signal and monitoring optical stimulation<br>HCImage, HCI-3570u (Hamamatsu Photonics, Shizuoka, Japan) for taking immunofluorescence images
Data analysis	MATLAB 2021a (MathWorks), Image Processing Toolbox, Signal Processing Toolbox, Statistic and Machine Learning Toolbox, Parallel Computing Toolbox

For manuscripts utilizing custom algorithms or software that are central to the research but not yet described in published literature, software must be made available to editors and reviewers. We strongly encourage code deposition in a community repository (e.g. GitHub). See the Nature Portfolio guidelines for submitting code & software for further information.

Data

Policy information about availability of data

All manuscripts must include a data availability statement. This statement should provide the following information, where applicable:

- Accession codes, unique identifiers, or web links for publicly available datasets
- A description of any restrictions on data availability
- For clinical datasets or third party data, please ensure that the statement adheres to our policy

All data supporting the findings of this study are available within the article and its supplementary files. Any additional requests for information can be directed to, and will be fulfilled by, the corresponding authors. Source data are provided with this paper. Extracellular potential data and a sample dataset were deposited to Figshare (https://doi.org/10.6084/m9.figshare.26880340).There are no restrictions to obtaining access to the primary data.

Research involving human participants, their data, or biological material

Policy information about studies with human participants or human data. See also policy information about sex, gender (identity/presentation), and sexual orientation and race, ethnicity and racism.

Reporting on sex and gender	n/a
Reporting on race, ethnicity, or other socially relevant groupings	n/a
Population characteristics	n/a
Recruitment	n/a
Ethics oversight	n/a

Note that full information on the approval of the study protocol must also be provided in the manuscript.

Field-specific reporting

Please select the one below that is the best fit for your research. If you are not sure, read the appropriate sections before making your selection.

☒ Life sciences ☐ Behavioural & social sciences ☐ Ecological, evolutionary & environmental sciences

For a reference copy of the document with all sections, see nature.com/documents/nr-reporting-summary-flat.pdf

Life sciences study design

All studies must disclose on these points even when the disclosure is negative.

Sample size	No sample-size calculations were performed. No statistical method was used to determine sample size. Experiment with 0.2 Hz optic stimulation were performed with n = 8 samples. Optical stimulation with 1 Hz were performed with n = 4 samples. All sample sizes were chosen based on standard practices in the field.
Data exclusions	When comparing BDRC in one burst versus successive bursts, directly and indirectly responding neurons were selected for reliability. The criteria were a response rate of at least 95% and a distance of less than 70 um from the stimulation site to the electrode. If more than one electrode met the criteria, one electrode with the highest absolute value of amplitude was selected.
Replication	All experiments with multiple biological replicates are indicated in the figure legends.
Randomization	This was not relevant to our study due to the absence of multiple conditions/groups to compare.
Blinding	No blinding was carried out due to the limited number of staff available to conduct these studies.

Reporting for specific materials, systems and methods

We require information from authors about some types of materials, experimental systems and methods used in many studies. Here, indicate whether each material, system or method listed is relevant to your study. If you are not sure if a list item applies to your research, read the appropriate section before selecting a response.

Materials & experimental systems

n/a	Involved in the study	
☐	☒	Antibodies
☒	☐	Eukaryotic cell lines
☒	☐	Palaeontology and archaeology
☐	☒	Animals and other organisms
☒	☐	Clinical data
☒	☐	Dual use research of concern
☒	☐	Plants

Methods

n/a	Involved in the study	
☒	☐	ChIP-seq
☒	☐	Flow cytometry
☒	☐	MRI-based neuroimaging

Antibodies

Antibodies used

mouse anti-MAP2 antibody (mouse, 1:250, MAB378, Clone AP20, Merck, Darmstadt, Germany)
rabbit anti-NeuN antibody (rabbit, 1:250, ab104225, Abcam, Cambridge, United Kingdom)
chicken anti-Beta III Tubulin antibody (chicken, 1:250, ab41489, Abcam)

Alexa Fluor 546 goat anti-rabbit IgG antibody (goat, 1:500, A-11010, Thermo Fisher Scientific)
Alexa Fluor 647 goat anti-mouse IgG H&L antibody (goat, 1:500; ab150115, Abcam)
Alexa Fluor 647 goat anti-chicken IgY H&L antibody (goat, 1:500; ab150171, Abcam)

Validation

anti-MAP2 antibody; https://www.merckmillipore.com/JP/ja/product/Anti-MAP2A-Antibody-AP20,MM_NF-MAB378
anti-NeuN antibody; https://www.abcam.co.jp/products/primary-antibodies/neun-antibody-neuronal-marker-ab104225.html
anti-Beta III Tubulin; https://www.abcam.co.jp/products/primary-antibodies/beta-iii-tubulin-antibody-ab41489.html

Animals and other research organisms

Policy information about studies involving animals; ARRIVE guidelines recommended for reporting animal research, and Sex and Gender in Research

Laboratory animals

Female Wistar rats (19th day of gestation) were obtained from Oriental Yeast Co., LTD., Tokyo, Japan.

Wild animals

No wild animals were used in this study.

Reporting on sex

Sex was not considered in this study because it do not affect the results.

Field-collected samples

This study did not involve samples collected from the field.

Ethics oversight

All procedures were approved by the University of Tokyo Animal Experiment Committee (approval number KA19-14).

Note that full information on the approval of the study protocol must also be provided in the manuscript.

Plants

Seed stocks

n/a

Novel plant genotypes

n/a

Authentication

n/a
